# Intranasal administration of poly-gamma glutamate induced antiviral activity and protective immune responses against H1N1 influenza A virus infection

**DOI:** 10.1186/s12985-015-0387-0

**Published:** 2015-10-06

**Authors:** Eun-Ha Kim, Young-Ki Choi, Chul-Joong Kim, Moon-Hee Sung, Haryoung Poo

**Affiliations:** Viral Disease Research Center, Korea Research Institute of Bioscience and Biotechnology, Daejon, Republic of Korea; College of Medicine, Chungbuk National University, Chengju, Republic of Korea; College of Veterinary Medicine, Chungnam National University, Daejeon, Republic of Korea; Department of Bio & Fermentation Convergence Technology, Kookmin University, Seoul, Republic of Korea; Infection and Immunity Research Center, Korea Research Institute of Bioscience and Biotechnology, Daejon, Republic of Korea

**Keywords:** Poly-gamma glutamate, Influenza virus, Antiviral activity, NK cell, Cytotoxic T cells

## Abstract

**Background:**

The global outbreak of a novel swine-origin strain of the 2009 H1N1 influenza A virus and the sudden, worldwide increase in oseltamivir-resistant H1N1 influenza A viruses highlight the urgent need for novel antiviral therapy.

**Methods:**

Here, we investigated the antiviral efficacy of poly-gamma glutamate (γ-PGA), a safe and edible biomaterial that is naturally synthesized by *Bacillus subtilis*, against A/Puerto Rico/8/1934 (PR8) and A/California/04/2009 (CA04) H1N1 influenza A virus infections in C57BL/6 mice.

**Results:**

Intranasal administration of γ-PGA for 5 days post-infection improved survival, increased production of antiviral cytokines including interferon-beta (IFN-β) and interleukin-12 (IL-12), and enhanced activation of natural killer (NK) cells and influenza antigen-specific cytotoxic T lymphocytes (CTL) activity.

**Conclusions:**

These results suggest that γ-PGA protects mice against H1N1 influenza A virus by enhancing antiviral immune responses.

## Background

Influenza A virus is an important human pathogen that continues to affect global health and cause pandemics sporadically, including the 2009 H1N1 influenza outbreak. Vaccination is the most economic and effective strategy to control influenza pandemics. However, because influenza vaccines target the virus’s surface antigen, they do not provide effective protection against influenza virus strains having variant antigens. Moreover, current anti-influenza drugs have several limitations and recent studies show that some human isolates of influenza A virus (H1N1) are resistant to antiviral drugs [1–3]. Because we cannot presage which influenza strain will cause the next epidemic or pandemic to ensure that vaccines target the most troublesome strain, we need to develop antiviral drugs that broadly control influenza by enhancing effective host immune responses.

During infection, viruses begin counteracting the host immune response comprising not only the innate but also the adaptive immune systems. For instance, infection of influenza virus blocks many features of antigen-presenting cells (APCs) such as dendritic cells (DCs) and macrophages, including production of the antiviral cytokine type I IFN [4]. Moreover, influenza virus-infected DCs fail to maturate costimulatory molecules and upregulate MHC class II molecules that are pivotal for antigen-specific, T-cell activation [5]. García-Satre et al. suggested that such modulation of the host immune response is a major mechanism whereby viruses create a favorable environment for their replication and persistence [6]. The hypothesis that prophylactic immunomodulators can be used to decrease influenza virus-induced mortality has been tested: The results suggest that immunomodulators, including CpG-oligonucleotide (CpG-ODN), can indeed initiate a rapid immune response characterized by antiviral and inflammatory cytokine production [7–9]. In fact, CpG-ODN have such strong immunostimulatory properties that pre-treatment with a single CpG-ODN dose of 1.5 or 2.5 μg provides partial protection to mice against lethal seasonal influenza virus.

Unfortunately, potent immunomodulator action is often correlated with increased toxicity. However, the immunomodulator poly-gamma glutamate (γ-PGA), which is produced by *Bacillus subtilis chungkookjang*, isolated from Chungkookjang, a traditional Korean fermented soybean food, is safe and edible [10]. Unlike other pathogen-associated molecular patterns, γ-PGA retains its water-soluble, biodegradable, and non-toxic properties [11, 12]. Previously, we identified this molecule as a potent inducer of the host immune response in in vivo tumor models [13, 14]. Recently, treatment with γ-PGA enhanced the type I interferon and protected them against highly pathogenic influenza A virus (H5N2) in the B6.A2G-Mx1 mouse model [15]. A biodegradable nanoparticles also have been demonstrated to be excellent immune stimulators with the potential to protect against viral infections [16]. Taken together, these investigations suggest that γ-PGA may be effective therapy for tumor and infectious diseases. On the basis of these results, we hypothesized that γ-PGA might be effective as an antiviral agent against influenza virus.

Because influenza viruses enter through a mucosal surface, preventing infection at the viral entry site by inducing mucosal immunity via intranasal immunization is an attractive strategy for influenza protection. In this study, we intranasally administered γ-PGA to C57BL/6 mice in an attempt to protect them from lung pathogenicity during infection with lethal doses of PR8 or CA04 H1N1 influenza A virus, strains known to induce severe immune pathogenicity, cause high mortality, and localize to pulmonary tissue in mice. We found that γ-PGA-treated mice had reduced mortality and pathogenicity during influenza infection. Moreover, intranasal administration of γ-PGA was found to effectively induce protective innate and CTL immune responses against both viruses. These findings suggest that γ-PGA may be an effective novel immunomodulator against influenza virus-induced pulmonary pathogenicity.

## Results

### γ-PGA induces antiviral activity against influenza A virus infection

**Fig. 1:**
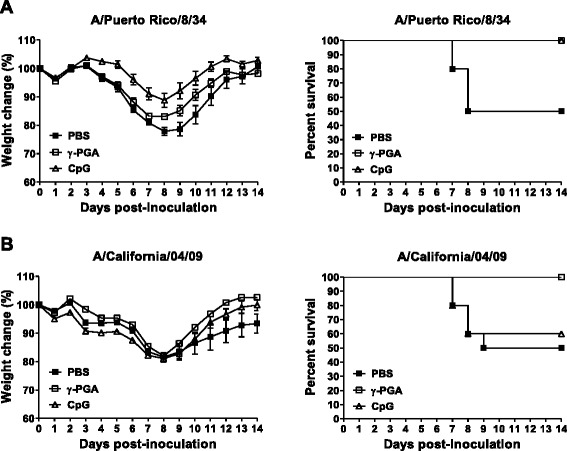
γ-PGA protects mice after a lethal dose of influenza A virus. C57BL/6 mice (*n* = 10 per group) were intranasally inoculated with 1 MLD_50_ of either (**a**) PR8 or (**b**) CA04 viruses. Mice were given either intranasal CpG-ODN 24 h prior to virus infection or intranasal γ-PGA 24 h post- inoculation (hpi). Body weight and survival were monitored daily for 14 days

To understand the therapeutic potential of immunomodulator treatment against influenza virus, we investigated the survival rate of mice after infection. We found that 50 % of the mice infected with PR8 alone were marked within 8 or 9 days of infection. In contrast, mice administered intranasal γ-PGA or CpG-ODN survived 14 days after PR8 virus infection, with only a slight loss of body weight (Fig. 1a). As shown in Fig. 1b, the survival rate of CA04-infected mice treated with γ-PGA administration (100 % survival) was higher than that of those treated with CpG-ODN (60 % survival). These results indicate that γ-PGA induced potent antiviral activity against influenza virus infection.

### γ-PGA induces viral clearance in mice with lethal influenza virus infection

**Fig. 2:**
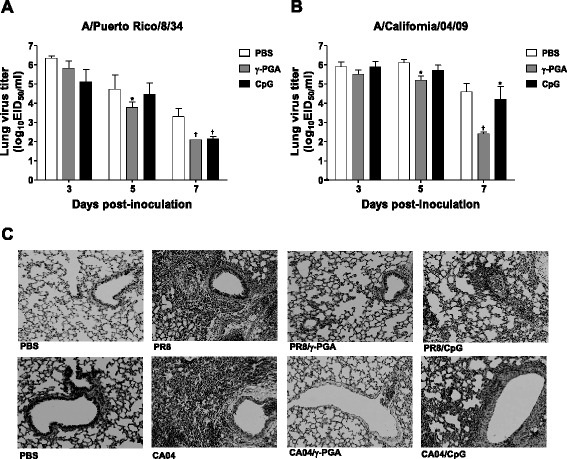
γ-PGA reduces the viral load and inflammation in mouse lung during influenza A virus infection. C57BL/6 mice (*n* = 5 per group) were intranasally inoculated with 1 MLD_50_ of either PR8 or CA04 viruses. Mice were given either intranasal CpG-ODN 24 h prior to virus inoculation or intranasal γ-PGA 24 h after infection. Mice were killed at 3, 5 or 7 days post-inoculation (dpi), and their lungs were collected for virus titer and histologic analyses. Titers of (**a**) PR8 and (**b**) CA04 viruses in the lung were measured at 5, 7, and 9 dpi. **c** H&E-stained, formalin-fixed lung samples of mice with or without immunomodulator treatment; PBS-only and virus-only negative controls are shown for comparison. Representative sections are shown (magnification, ×100). † indicates *p* < 0.01 compared to the control group; * *p* < 0.05 relative to the control group

To assess the immunomodulators’ effects on pulmonary pathology in response to influenza virus infection, we determined the virus titers in the lungs of influenza-infected mice. At 5 dpi, a difference in the viral load in the lung could be observed between γ-PGA- and CpG-ODN-treated mice infected with either virus (Fig. 2a and b). Compared to PBS treatment, γ-PGA significantly decreased virus titers in the lungs of CA04 virus-infected mice, whereas no significant difference in viral load was observed between CpG-ODN- and PBS-treated mice infected with either virus. Compared to PBS-treated controls, γ-PGA- and CpG-ODN-treated mice each had significantly lower viral loads in the lung at 7 dpi (*p* < 0.01, *p* < 0.05, respectively; Fig. 2a and b).

Next, we evaluated the extent of the pathologic changes in the lungs of influenza virus-infected mice treated with immunomodulators. Typically, influenza A virus replication is accompanied by infiltration of immune cells in the lung tissue of the infected host [9]. Consistent with published data, we observed induction of widespread inflammatory processes in influenza virus-infected mice not treated with immunomodulators. As expected, no histologic alterations indicative of severe inflammation or disruption of the membrane barrier were observed in the lung tissue of virus-infected mice given γ-PGA (Fig. 2c). Furthermore, only mild histopathologic changes were detected in the lungs of a PR8-infected mouse given CpG-ODN. However, lungs of CA04 virus-infected mice given CpG-ODN had considerably more bronchial cell infiltrates and peribronchial inflammation. Collectively, these results show that intranasal administration of γ-PGA could prevent histopathologic alterations in the lungs and protect mice against lethal influenza virus infection.

### γ-PGA enhances activation of innate immune cells in influenza-infected mice

**Fig. 3:**
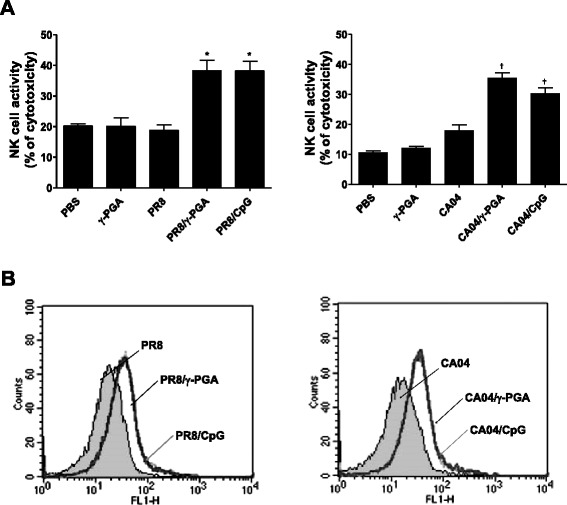
γ-PGA promotes NK cells’ cytotoxicity and enhances DCs’ maturation in influenza virus-infected mice. C57BL/6 mice (*n* = 5) were infected with PR8 and CA04 influenza viruses at 1 LD_50_ per mouse. After 24 h, mice were intranasally administrated with 100 μg of γ-PGA and 5 μg of CpG-ODN administrated prior to virus infection for 24 h. **a** Lung cell suspensions were magnetically separated to purify NK cells by positive selection using the CD49b antibody. Purified NK cells were mixed with target cells at an effector cell to target cell ratio of 50:1, and specific lysis values were quantified by performing lactate dehydrogenase release assays. **b** Cell suspensions were stained with anti-mouse CD86 antibody. To avoid nonspecific counting, cells were gated with anti-mouse CD11c + antibody and then DCs’ maturation was measured by flow cytometry. In each overlay, unfilled histograms represent the expression of maturation marker on lung DCs. Data are representative of two independent experiments

In the early phase of influenza virus infection, host immune responses include inflammatory cytokines, with immune cells such as NK cells and DCs playing a critical role in host defense [17, 18]. Norton et al. suggest that immunomodulators such as CpG-ODN and cholera toxin strongly protect against viral infections by promoting the innate immune response [9]. We, therefore, examined whether γ-PGA alters immune cell activation in the lungs during influenza virus infection. As expected, cytotoxicity activity in purified lung NK cells from either γ-PGA- or CpG-ODN-treated mice was approximately 2.5-fold higher than that of cells from untreated mice (Fig. 3a).

Next, we assessed γ-PGA’s ability to induce maturation of DCs in influenza virus-infected mice by using FACS analysis to measure the expression of maturation marker CD86 in lung DCs. Both γ-PGA- and CpG-ODN-treated mice had higher CD86 expression than untreated mice did (Fig. 3b). In addition, we confirmed that other DC maturation markers such as CD40 and CD80 are expressed in higher amounts in lung DCs from γ-PGA-treated mice than in those from untreated mice (data not shown). Taken together, these results show that intranasal γ-PGA promotes an innate immune response in the lung after activating NK cells’ cytotoxicity and DCs’ maturation during influenza virus infection.

### γ-PGA induces cytokine production in mice infected with influenza virus

**Fig. 4:**
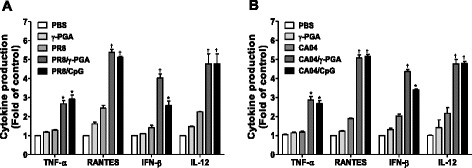
. γ-PGA induces cytokine production in mice infected with influenza virus. C57BL/6 mice (*n* = 5) were infected with PR8 and CA04 influenza viruses at 1 LD_50_ per mouse. After 24 h, mice were intranasally administrated with 100 μg of γ-PGA and 5 μg of CpG-ODN administrated prior to virus infection for 24 h. Two days later, mice sacrificed and lung tissue homogenized. Cytokine production in lung homogenates of (**a**) PR8 and (**b**) CA04 virus infected mice was analyzed: TNF-α, RANTES, IFN-β and IL-12 by ELISA. Data are representative of two independent experiments. † indicates *p* < 0.01 compared to the virus infection group; * *p* < 0.05 relative to the virus infection group

Because cytokines trigger innate and adaptive immune responses, including APC maturation and induction of cytotoxic NK cells and CTLs, they can protect against virus infection [19, 20]. Österlund et al. suggest that the pandemic 2009 H1N1 influenza virus induces a weak innate immune response, producing only small amounts of antiviral and inflammatory cytokines, particularly IFN-α, IFN-β, and TNF-α [21]. To determine whether γ-PGA affects cytokine production during influenza virus infection, we examined the amount of cytokines in lung homogenates by using ELISA. Consistent with previous results [7], CpG-ODN induced increased cytokine production in lung homogenates during influenza virus infection (Fig. 4). TNF-α, RANTES, IL-12, and IFN-β production levels were significantly higher in γ-PGA-treated mice than in untreated mice (Fig. 4). Therefore, γ-PGA also induced increased cytokine production in lung homogenates during influenza virus infection.

### γ-PGA promotes influenza virus-specific CTL activity in mice infected with influenza virus

**Fig. 5:**
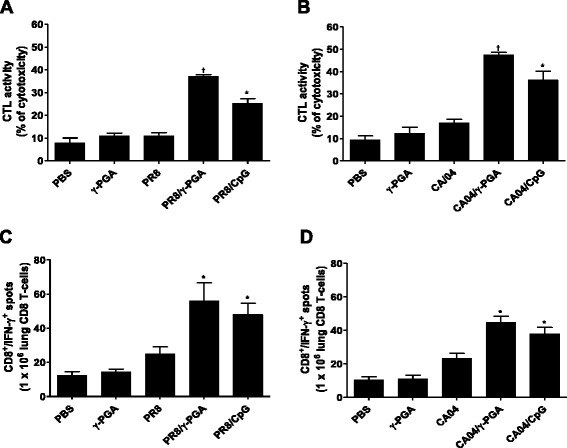
γ-PGA promotes influenza-specific CTL activity in mice with infected influenza virus. C57BL/6 mice (*n* = 5) were infected with PR8 and CA04 influenza viruses at 1 LD_50_ per mouse. After 24 h, mice were intranasally administrated with 100 μg of γ-PGA and 5 μg of CpG-ODN administrated prior to virus infection for 24 h. Nine days after infection, purified CD8+ T cells isolated from single-cell, lung suspensions of (**a**) PR8 and (**b**) CA04 virus infected mice were used as follows. To determine CTL activity, purified CD8+ T cells were mixed with target cells at an effector cell to target cell ratio of 50:1, and specific lysis values were quantified by performing lactate dehydrogenase release assays. For the ELISPOT assay, purified CD8+ T cells of (**c**) PR8 and (**d**) CA04 infected mice were stimulated with UV-inactivated influenza viruses and incubated for 72 h. Data are representative of two independent experiments with four to five replicate wells per group. *Bars* signify that test groups were significantly different from virus-alone groups when analyzed by *t*-test († indicates *p* < 0.01 compared to the virus infection group; *, *p* < 0.05 relative to the virus infection group)

Influenza virus-specific, CD8^+^ T cell-mediated immunity is a critical component of the host response after infection with H5N1, H3N2, or H1N1 virus strains [22, 23]. The capacity of CD8^+^ T cells to produce multiple effector cytokines such as IFN-β and TNF-α has also emerged as a positive correlate of effective CTL immunity after virus infection [24], suggesting that CD8^+^ T cells contribute to the primary mechanism of virus clearance. To determine whether γ-PGA elicits effective, influenza-specific CTL activity, mice were treated with γ-PGA for 7 days after infection with either CA04 or PR8 virus. On day 9, the cytotoxic activity of lung CD8^+^ T cells was assayed. As shown in Fig. 5a and b, lung CD8^+^ T cells from γ-PGA-treated mice had high levels of CTL activity against both influenza viruses. In addition, the influenza-specificity of the IFN-γ-expressing CD8^+^ T cell response was observed after stimulation with either UV-inactivated CA04 or PR8 virus. Consistent with the CTL activity results, the number of CD8^+^/IFN-γ-secreting cells was significantly higher in γ-PGA treated-mice than in untreated mice (Fig. 5c and d), with CpG-ODN inducing responses similar to those induced by γ-PGA. Taken together, our data clearly show that γ-PGA plays a crucial role in inducing the anti-influenza cytotoxic activity of CD8^+^ T cells in the lung.

### The antiviral effects of γ-PGA are dependent on CD8^+^ T cells in CD4^+^ or CD8^+^ T cell depleted mice models

**Fig. 6:**
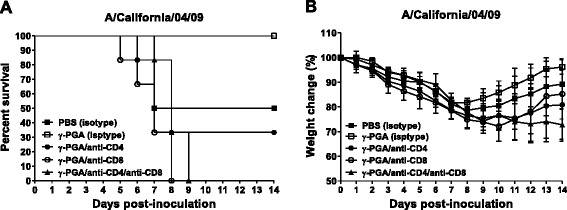
The depletion of CD8^+^ T cells eliminates the antiviral effect of γ-PGA against influenza A virus infection. Anti-CD4 (GK1.5), anti-CD8 (53.6.72), and isotype control antibodies were injected into mice before and after challenge. **a** Body weight loss and (**b**) survival were monitored for 14 days

CD4^+^ and CD8^+^ T cells are both reported to play roles in clearing primary influenza virus infections [25]. Eichelberger et al. found that mice depleted of CD8^+^ T cells cleared virus similarly to normal mice [26], casting some doubt on the relative importance of CD8^+^ T cells in host defense against viral infections. To confirm which subsets of T lymphocytes are important for protection against lethal CA04 virus infection, we performed in vivo antibody depletion experiments. As shown in Fig. 6, preventive antiviral effects were abrogated through CD8^+^ and CD4^+^ plus CD8^+^ T cell depletion in the γ-PGA-treated mice. Body weight loss of the mice in these two groups was greater than that in mice in the isotype control group (Fig. 6a), and none of the mice survived (Fig. 6b). Depletion of CD4^+^ T cells also decreased the level of protection in γ-PGA-treated mice but not to the same degree as that seen with CD8^+^ T cell depletion (Fig. 6b). In addition, γ-PGA-treated mice that received isotype control antibody injections also suffered weight loss; but, they recovered rapidly after day 9, and all the mice survived (Fig. 6). These results indicate that CD8^+^ T cells were essential for the anti-influenza effect generated by γ-PGA.

## Discussion

The emergence of highly contagious influenza virus strains, such as the new pandemic H1N1 influenza, represents a serious threat to global human health, and influenza virus vaccines do not protect against antigenically drifted strains that frequently appear to cause seasonal influenza outbreaks. Given the emergence of drug-resistant strains and limited protection capability, a great need exists to develop strategies to protect against the next pandemic influenza. The current study evaluated the antiviral efficacy of intranasal administration of γ-PGA to prevent lethality in mice infected with H1N1 influenza A viruses.

Norton et al. reported that intranasal immunomodulators induce a local immune response that results in decreased mortality upon influenza A virus infection [9]. In the present study, we showed that intranasal administration of γ-PGA shortly after viral infection could inhibit influenza virus replication, leading to a significant reduction in pulmonary viral titers and an improved survival rate of infected mice. Recently, Okamoto et al. reported that intranasal vaccination with γ-PGA nanoparticles containing influenza HA protein enhances protection against influenza virus infection [27]. Taken together, although therapeutic evaluation in humans is required, these findings indicate a therapeutic utility for γ-PGA when used as an anti-influenza virus drug.

To develop optimal drugs against the influenza viruses, it is important to understand their mechanisms of action on the immune system [28]. Host innate immune response is the first line of protection against infection by viruses and is essential in local control of invading microbes. During influenza infection, DCs are necessary to induce an immune response that clears the virus from the lungs [29, 30]. In addition, influenza virus is able to evade host immunity by attenuating NK cell lysis of influenza virus-infected cells [30, 31]. Österlund et al. reported that pandemic H1N1 (A/Finland/553/09) virus and seasonal H1N1 virus (A/Brisbane/59/07) induce a relatively weak innate immune response in macrophages and DCs, as evidenced by a poor expression of antiviral and inflammatory cytokine genes [21]. IFN-β can inhibit the replication of a wide variety of viruses, which contributes to key effector molecules of the immune response to viruses [32]. IL-12 and RANTES are also important cytokines involved in cellular immunity, which can potently stimulate cytotoxic T cells as well as NK cells’ activation [33–35]. Therefore, cytokines are promising of antiviral drugs for enhancing the host immune response against influenza virus infection. We found that cytokine production levels and activation of innate immune cells in lung samples from γ-PGA-treated mice were higher than those from untreated controls. In addition, we also observed a significantly higher number of activated NK1.1+ cells in total lung cells from γ-PGA-treated mice than in those from untreated mice (data not shown). Thus, our findings suggest that intranasal γ-PGA promotes the innate immune response during virus infection, protecting against influenza.

Cell-mediated immune responses play a crucial role in protecting the host from invading pathogens [36, 37]. Virus clearance requires strong Th1-polarized immune responses characterized by IFN- γ production and CTL responses in the systemic compartment [38]. Moreover, earlier studies show that both CD4^+^ and CD8^+^ T cells contribute equally to protection against pandemic H1N1 influenza virus [24, 25]. In accordance with these reports, we observed that in vivo depletion of CD4^+^ and CD8^+^ T cells completely abrogated antiviral activity in γ-PGA-treated mice, with γ-PGA having no antiviral effects in CD8^+^ T cell-deficient mice. Our data clearly showed that γ-PGA increases the cytotoxic activity of CD8^+^ T cells in the lung against H1N1 influenza A viruses. Hanna et al. reported the precise contribution of NK cells to induction of adaptive immunity during influenza infection of the lung [39]. Moreover, other studies have shown that depletion of NK1.1+ cells decreases the induction of virus-specific CTL activity and increases influenza disease severity during influenza virus infection in mice [40]. The major function of DCs during an antiviral immune response is to process and present viral antigenic peptides in the draining lymph nodes to rare, antigen-specific T cells [41]. Additionally, cytokines such as IL-12 are key molecules that trigger the adaptive immune responses (including of DCs maturation, differentiation of Th1 and Th2 cells, and induction of cytotoxic NK cells and CTLs), resulting in protective layers against virus infection [42, 43]. Taken together, our results indicate that γ-PGA enhances influenza-specific CD8^+^ T cell immunity, increasing cytokine production and activation of antigen-presenting cells. However, the molecular mechanism by which γ-PGA provided complete protection against influenza virus remains unknown.

## Conclusions

In our study, we confirmed that γ-PGA enhances influenza-specific CD8+ T cell immunity, increasing cytokine production and activation of antigen-presenting cells. We also showed that administration of γ-PGA significantly improved the survival rate and virus inhibition of mice from infections of PR8 and CA04 viruses. Although further mechanism studies are needed, we showed that intranasal γ-PGA induced a higher influenza-specific CTL response and innate immune response than did CpG-ODN after influenza H1N1 virus infection. In light of this evidence, we suggest that γ-PGA may be a more potent immunomodulator against H1N1 influenza A virus infection than CpG-ODN.

In summary, we report for the first time that intranasal administration of γ-PGA generated significant antiviral activity in H1N1 influenza A virus-infected mice. These novel findings indicate that γ-PGA mediates anti-viral immunity via the induction of antigen-specific CTL activity, suggesting that this molecule could be a good candidate for development as a novel immunomodulator for anti-influenza treatment.

## Methods

### Mice, cells, and viruses

Six-week-old female C57BL/6 (H-2^b^) mice were purchased from KOATECH (Pyeongtaek, Korea) and housed in specific pathogen-free conditions at Korea Institute of Bioscience and Biotechnology (KRIBB) in Daejeon, Korea. Mouse experiments were approved by KRIBB’s Institutional Review Board. The mouse lymphoma cell line EL-4 (H-2^b^) was obtained from the Korea Cell Line Bank (Seoul, Korea) and maintained in complete Dulbecco’s modified Eagle’s medium (DMEM; Hyclone; UT, USA) supplemented with 10 % fetal bovine serum (FBS; Hyclone). Both A/Puerto Rico/8/1934 (PR8) and A/California/04/2009 (CA04) H1N1 influenza A viruses were grown for 2 days at 37 °C in the allantoic cavities of 10-day-old fertile chicken eggs. Virus stocks were propagated in the allantoic cavity of 10-day-old, embryonated, and specific pathogen-free hen’s eggs at 37 °C. Clarified allantoic fluid was aliquoted and immediately frozen at −80 °C.

### Influenza virus infection

Mice were lightly anaesthetized and challenged intranasally with 50 % of the mouse lethal dose (MLD_50_) of PR8 or CA04 influenza A viruses in a total volume of 30 μl. After infection, mice were monitored daily for morbidity (i.e., body weight loss) and survival for up to 14 days post-infection (dpi). Individual body weights were recorded for each group on various dpi. The mice having more than 20 % of body weight lost were considered to have reached the experimental endpoint and were humanely killed.

To determine the titer of infectious virus, lung samples of 3 mice per group were collected at 3, 5, and 7 dpi. Lung samples were homogenized in 1 ml of sterile PBS containing penicillin G (200,000 U/ml), streptomycin (40,000 U/ml), polymyxin B (20,000 U/ml), and gentamicin (5 mg/ml). The titers of clarified lung tissue homogenates were determined by calculating the standard 50 % egg infectious dose (EID_50_); the detection limit was 0.75 log_10_ EID_50_/ml.

### Immunomodulator treatment

Because CpG-ODN has been demonstrated to be an excellent immunomodulator with the potential to protect against influenza virus infection [7], it was used as the positive control for our experiments to test the anti-influenza efficacy of γ-PGA immunomodulator treatment. CpG-ODN was purchased from InvivoGen (SD, USA), and γ-PGA was kindly provided by Bioleaders Corporation (Daejeon, Korea). Mice were infected with either CA04 or PR8 (1 MLD_50_), and the immunomodulators were used as follows: γ-PGA (100 μg in 20 μl endotoxin-free PBS [Sigma; MO, USA]) was intranasally administered to mice for 7 days starting 24 h after virus infection or CpG-ODN (5 μg in endotoxin-free PBS) was intranasally administered once 24 h prior to virus infection. Control mice were administered an equal volume of endotoxin-free PBS.

### Histopathology

At 5 dpi, fresh lung tissues were collected from PBS-treated, CpG-ODN-treated, and γ-PGA-treated mice (*n* = 3 per group), fixed in 10 % neutral buffered formalin and then dehydrated, embedded in paraffin, and cut into 4 μm-thick sections. The sections were stained with hematoxylin and eosin (H&E) and examined by using an Olympus TH4-200 System Microscope attached to a DP72 Microscope Digital Camera. The captured images were analyzed by using DP2-BSW software (Ver.2.1, OLYMPUS).

### Flow cytometry analysis

Single-cell suspensions were prepared from mouse lung by using type II collagenase and DNase treatment. In brief, whole lung tissue was removed, minced in collagenase digestion solution (10 ml PBS containing 20 μg/ml type II collagenase [Roche, NJ, USA] and 100 μg/ml DNase I [Roche]), and incubated at 37 °C for 30 min. Lung tissue pieces were meshed through a 40-μm cell strainer (Falcon) before red blood cells in the supernatant were lysed by using red blood cell-lysis buffer (Sigma). To measure the population of lung immune cells, approximately 1 × 10^6^ cells were stained in 100 μl FACS buffer (PBS containing 2 % FBS [*v*/*v*] and 0.02 % sodium azide [*v*/*v*]) with FITC-conjugated anti-mouse CD40, CD80, and CD86 antibodies. To avoid nonspecific counting, cells were gated with either anti-mouse CD49b or anti-mouse CD11c^+^ antibody. All antibodies were purchased from BD Biosciences (CA, USA). Data were acquired on a BD FACSCalibur (BD Biosciences) and analyzed by using the CELLQuest Pro software (BD Biosciences).

### Quantification of cytokines

To count cytokines, mouse lung tissue was harvested and homogenized in 2 ml of tissue protein extraction reagent (Pierce: IL, USA) containing protease inhibitor cocktail (Roche). The homogenate was centrifuged at 16,000 g for 20 min at 4 °C before the supernatant was harvested. The amounts of the following cytokines were analyzed: IL-1β, IL-4, IL-6, IL-12p40, IL-15, IFN-γ, tumor necrosis factor-α (TNF-α), and regulated on activation normal T-cell expressed and secreted (RANTES). The concentration of IFN-β in the homogenate was determined by using an IFN-β ELISA kit (PBL Laboratories; NJ, USA).

### Cytotoxicity assay

To isolate the CD8^+^ T cells and NK cells, total lung cells were first digested in collagenase digestion solution. Both cell types were magnetically separated via negative selection with a CD8^+^ T-cell isolation kit (Miltenyi Biotec; CA, USA) and positive selection with a CD49b (DX5) kit (Miltenyi Biotec). The cell-mediated cytotoxicity assay was performed using the EL-4 cells as target cells [44] and quantified by using the lactate dehydrogenase (LDH)-release assay kit according to the instructions (Promega; CA, USA). In brief, lung NK cells and CD8^+^ T cells were mixed with target cells at an effector cell to target cell ratio of 50:1 and incubated at 37 °C for 4 h. To determine the CD8^+^ T cells’ cytotoxicity, target cells were stimulated with the 50 % tissue culture-infective dose (TCID_50_) of UV-inactivated virus for 24 h. Each sample was incubated with 50 μl of substrate at room temperature for 30 min, and optical density (OD) values were measured at 490 nm wavelength by using the VICTOR3 1420 multilabel counter plate reader (Perkin-Elmer; MA, USA). The cytotoxicity percentage was calculated by using the following formula: (mean experimental OD value–mean spontaneous OD value)/(mean maximal OD value–mean spontaneous OD value) × 100.

### IFN-γ ELISPOT assay

Ninety-six-well filtration plates (Millipore, MA, USA) were pre-coated with anti-mouse IFN-γ antibody (5 μg/ml; BD Biosciences) overnight at 4 °C. After the plates were washed four times with PBS and then blocked with RPMI 1640 medium (Gibco), CD8^+^ T cells (1 × 10^5^ cells in 100 μl of medium) were added to each well of the filtration plates along with UV-inactivated influenza virus (1 TCID_50_) [45] in 100 μl of medium). The plates were incubated for 72 h, washed with PBS containing 0.5 % Tween-20 (PBS-T), and incubated with biotin-conjugated anti-mouse IFN-γ antibody (1 μg/ml) for 2 h at room temperature. After 5 washes with PBS-T, diluted horseradish peroxidase (HRP)-conjugated streptavidin antibody (1:200; BD Biosciences) was added to the each well and incubated at room temperature for 1 h. The cells were washed 5 times with PBS-T; ELISPOT AEC substrate (100 μl; BD Biosciences) was added to develop the reaction, and then the reaction was stopped by washing the cells with double-distilled water. The spots were counted automatically by using the ELISPOT CTL-ImmunoSpot S5 UV Analyzer (Cellular Technology; OH, USA).

### CD4^+^ and CD8^+^ T cell depletion

For T-cell depletion studies, mice were immunized with 100 μg of γ-PGA 5 days after influenza virus inoculation. Groups of mice were then injected intraperitoneally with 100 μg anti-CD4 antibody (clone GK1.5; BioLegend), 100 μg anti-CD8 antibody (clone 53.6.72; BioLegend), and 100 μg of both IgG2b isotype control antibodies (BioLegend). Antibodies were injected every other day three times before and twice at 3 days and 7 days after inoculated with a MLD_50_ of CA04 virus (5.5 EID_50_/ml per mouse). The positive control group was not injected with antibodies but was γ-PGA-treated after challenge. After virus infection, mice were monitored daily for weight loss and survival.

### Statistical analyses

The analyses were done by using GraphPad Prism version 5.00 for Windows (GraphPad Software, CA, USA). *P* values of less than 0.05 (*p* < 0.05) were considered to be statistically significant.

### Ethics statement

All animal experiments were approved by the Institutional Animal Use and Care Committee of the Korea Research Institute of Bioscience and Biotechnology and were performed in accordance with the Guide for the Care and Use of Laboratory Animals published by the US National Institutes of Health.
